# Chemical Ecology of Host- and Mate-Finding in the Cypress Bark Beetle *Phloeosinus aubei*, with Notes on Congeneric Species

**DOI:** 10.3390/insects17010107

**Published:** 2026-01-16

**Authors:** Gábor Bozsik, Armin Tröger, Stefan Schulz, Michael J. Domingue, Gábor Szőcs

**Affiliations:** 1 Plant Protection Institute, Centre for Agricultural Research, Hungarian Research Network (HUN-REN), 2462 Martonvásár, Hungary; bozsik.gabor@atk.hun-ren.hu; 2 Institute for Organic Chemistry, University of Hamburg, 20148 Hamburg, Germany; armin.troeger@uni-hamburg.de; 3 Institute of Organic Chemistry, TU Braunschweig, 38092 Braunschweig, Germany; stefan.schulz@tu-braunschweig.de; 4 Department of Entomology, Kansas State University, Manhattan, KS 66506, USA

**Keywords:** *Phloeosinus* spp., bark beetle, scale-leaved conifers, host plant kairomone, aggregation pheromone

## Abstract

Recent intensive research on the cypress bark beetle, *Phloeosinus aubei* was prompted because of its invasion of Central Europe that caused serious damage to scale-leaved conifer ornamental trees. This dynamic also increased the risk of accidental introduction into North America. In contrast to other historically well-studied bark beetles infesting spruce, pine or broad-leaf trees, intense study of the pheromones and host plant kairomones of bark beetles associated with cupressaceous trees has only begun in the past decade. This highly specialized clade is represented by the genus *Phloeosinus*. The pressing need for semiochemical-baited traps demands the identification of behavior-modifying compounds. This challenge involves unraveling the various stimuli interacting in the complex communication system to reveal the composition of signal bouquets and the absolute configuration of their components capable of evoking behavior responses. In this short overview we describe the recent research results on host-finding and intraspecific chemical communication of *P. aubei*, with a short outlook on the species of this genus.

## Simple Summary

The Mediterranean cypress bark beetle (CBB), *Phloeosinus aubei*, is a serious xylophagous pest of scale-leaved conifers of the Cupressaceae family. Its increasing silvicultural importance and accelerated spread, favored by climate change and driven by international timber trade, has brought it into the focus of chemoecological research. The latest investigations on host plant volatiles and intraspecific signaling have paved the way for the development of attractant traps for monitoring and mass trapping. In this review, we provide insight into the complex chemical communication system of *P. aubei*. First, host selection by the females occurs using kairomones. Then aggregation pheromones attract more conspecifics, initially produced by the pioneering females and reinforced by both sexes as mass colonization ensues. Finally, an inhibitory signal is emitted to limit population density.

## 1. Introduction

The cypress bark beetle, *Phloeosinus aubei* (Perris, 1855) (Coleoptera: Curculionidae: Scolytinae), has attracted increasing attention over the past decades due to its northward expansion from the Mediterranean region into Central and Northern Europe (see references in Section 3.3). This invasion has also drawn attention to the previously understudied clade of bark beetles associated with scale-leaved conifers (Cupressaceae), represented by several species of the genus *Phloeosinus*.

While bark beetles specialized to pin-leaved conifers have been intensively studied, leading to a detailed understanding of their host- and mate-finding mechanisms and enabling the development of kairomone- and pheromone-based trapping tools, comparable knowledge for *Phloeosinus* species has only emerged recently. Closing this gap is increasingly critical given the economic damage caused by *P. aubei* in ornamental tree nurseries and urban green areas.

Here we review the latest findings on the chemical ecology of bark beetles infesting cupressaceous trees, focusing primarily on *P. aubei* and, where relevant, on other species of the genus.

## 2. Chemical Ecology of Bark Beetles: General Concepts and Historical Background

To place recent findings on *Phloeosinus* species in a broader context, the following section briefly summarizes the general principles and historical development of bark beetle chemical ecology.

Phytophagous insects specialized to a narrow host plant range typically rely on specific chemical cues to locate suitable hosts, with host plant volatiles playing a crucial role in long-range orientation. Following early advances in pheromone research [1], research in coleopteran chemical ecology rapidly expanded, largely driven by the economic importance of bark beetles in forest ecosystems.

Initial work within this field focused on bark beetle species (Coleoptera: Curculionidae: Scolytinae) associated with pin-leaved conifers (Pinaceae). Early studies demonstrated that host plant volatiles are key attractants for these insects [2], and that terpenes such as α-pinene and camphene emitted by ponderosa pine and Douglas fir elicit behavioral responses in specialized bark beetle species [2,3,4]. It soon became evident that host-derived cues alone do not fully explain host colonization, as beetle-produced intraspecific chemical signals interact with plant volatiles to coordinate mass attacks [5].

The first Scolytinae identified bark beetle pheromones were produced by the host-selecting sexes and originated in the hindgut [6], eliciting aggregation behavior in both males and females. Compounds such as ipsenol, ipsdienol and *cis*-verbenol were shown to be emitted by pioneer males of *Ips paraconfusus* Lanier, 1970 [3], while *exo*-brevicomin was identified from the frass of female western pine beetles, *Dendroctonus brevicormis* LeConte, 1876 [6,7]. Subsequent research revealed that secondary attacking sexes also contribute pheromones that promote mass aggregation and regulate sex ratios [8]. In later stages of colonization, inhibitory signals are often released to prevent overpopulation [9], with verbenone produced by *Dendroctonus* males being a well-known example [10]. The multi-component chemical signals guiding bark beetles within forest habitats toward suitable host trees have since been described in detail [11]. Furthermore, the importance of male stridulation in the mating sequence has also been demonstrated [12,13].

Species specificity of bark beetle pheromones is frequently achieved through the diastereomer or enantiomer composition of chiral components, as well as through synergistic interactions among multiple compounds [14,15,16]. For instance, *trans*-verbenol is produced by *Dendroctonus* females, whereas *Ips* males and females enantioselectively oxidize (−)-α-pinene to (4*S*)-*cis*-verbenol [17,18]. Continued research led to the identification of increasingly specific pheromone structures, including bicyclic acetals such as brevicomin and spiroacetals such as chalcogran [19,20,21,22].

## 3. Biology of Bark Beetles Associated with Cupressaceae

### 3.1. Taxonomy, Host Plants, Pest Status and Original Distribution

The few bark beetle species that are specialized on scale-leaved conifers (Cupressaceae) are confined to the genus *Phloeosinus*. Species of this genus typically develop in the Mediterranean region on various native conifers, of the plant genera *Cupressus*, *Chamaecyparis*, *Cedrus*, *Thuja*, and *Juniperus*. In 1956, Zocchi published a review on the biology and taxonomy of the genus describing 89 species that occur worldwide [23]. A comprehensive taxonomical overview by [24] provides the key for identifying eight central- and west-palearctic species, belonging to the *Phloeosinus* Chapuis, 1969 genus. This genus of the tribe Phloeosinini Nüsslin, 1912 [24] is the only one developing in either scale-leaved conifers or cedars (*Cedrus* spp.). *Hypothenemus eruditus* Westwood, 1834 is a highly polyphagous bark beetle, and although it primarily occurs on a wide range of hosts, it has also been recorded on Cupressaceae, including *Cryptomeria japonica* D. Don [25]. Löbl and Smetana listed thirty palearctic *Phloeosinus* species [26]. And among these, *Phloeosinus laricionis* Faccoli & Sidoti, 2013, newly described by the authors, is the only that infests pines [27]. Similarly, of 30 known North American *Phloeosinus* species, only *Phloeosinus pini* Swaine, 1915, attacks pine trees [28,29].

Eight species are known to exist in Europe and Mediterranean regions of northern Africa and western Asia. Of these, cypress bark beetle, *Phloeosinus aubei* (Perris, 1855) is the most economically important. Four species, including *P. aubei*, have been described in Italy, all pests of *Cupressus* or other members of the Cupressaceae family [23,24]. In Israel, *Phloeosinus armatus* Reitter, 1887 and *P. aubei* have been reported as important pests of Cupressaceae [30,31]. In Tunisia, *P. aubei* is the dominant *Phloeosinus* species [32]. In Greece, *P. aubei* (syn. *P. bicolor* Brullé, 1832) is an emerging pest that threatens urban trees, tree nurseries and Cupressaceae forests [33].

The list of *Phloeosinus* species present in Europe and their invasive status are shown in Table 1.

### 3.2. Life Cycle

In the Mediterranean region, *P. aubei* is multivoltine. Newly emerged adults feed on the host tree, then the females as the pioneer sex initiate carving new breeding galleries [23,30,44,45]. For example, three to four annual generations have been reported in Israel [32], two to four in Greece [32] and two in Tunisia [31].

In the newly colonized European regions, *P. aubei* has adapted to Atlantic or continental climates. In Hungary, for example, *P. aubei* has only one annual generation, albeit with two flight periods. The adults of the new generation emerge between late summer and autumn and hibernate to overwinter. For this purpose, they construct a different type of gallery, inhabited by a single beetle and located at the junctions of thin twigs near to the apical part of this year’s shoot tips. Maturation feeding on foliage and, occasionally, on bark was observed before and after overwintering. In spring, they leave their overwintering tunnels to mate [45].

### 3.3. Area Expansion and Pest Status of Invasive Species in Europe

Many insect species have a remarkable ability to adapt to new habitats. The trigger for migration is often climate change, making new regions adjacent to the original habitat suitable for survival. In addition, invasive species are frequently introduced accidentally through international trade and travel. The spread of *Phloeosinus* bark beetles across Europe is probably primarily driven by climate change. However, transport of ornamental evergreen trees could also contribute to this process. The colonization of new regions by alien insect species is often very apparent through the immediate damages they cause to cultivated or wild plants. However, they can remain cryptic for years, even decades, before a population spike reveals a successful settlement.

For example, *P. aubei* was recognized in Hungary as early as the 1950s [46]. However, the first alerts about economic damages came much later, in the early 1990s, from southwestern Hungary [47,48]. This suggests that the beetles migrated to Hungary following the Illyrian insect migration route east of the Alps [49] and then colonized the country from southwest to northeast [50].

Due to the wide original distribution area of *P. aubei*, spanning from the Caucasus and Asia Minor to the entire Mediterranean region [51], it is highly probable that its migration to Western and Central Europe occurred along various routes. Today, there are established populations in the Netherlands [34], southern Hesse and eastern Germany [52,53], Romania [54], and the Czech Republic [35]. The introduction to Britain in 1989 may have occurred through the trading of plants [55].

This illustrates the high adaptability of *P. aubei* to different climates. Most probably, *P. aubei* will continue to spread, threatening ornamental conifers and junipers in northern and temperate regions of Europe, Asia, and North America.

### 3.4. Risk of Phloeosinus Bark Beetles in North America

*Phloeosinus punctatus* LeConte, 1876, is a well-known pest of North American Cupressaceae species. Species such as giant sequoia, *Sequoiadendron giganteum* [Lindl.], are known to be susceptible to the pest during periods of drought [56]. The Oriental cypress bark beetle, *P. armatus*, has also been discovered in southern California in ornamental cypress trees, with *P. aubei* considered to be another looming threat to the many Cupressaceae species and cultivars in the state [57]. Dispersal routes for invasive *Phloeosinus* species in North America are thus well known, with the Cupressaceae species that contribute to the large and diverse forests along the western coast of north America being at significant risk.

## 4. Chemical Ecology of *Phloeosinus aubei*

In contrast to the comprehensive knowledge about the chemical ecology of diverse bark beetles in conifers [58], the communication modalities of bark beetles in scaled-leaved conifers have only been studied in detail in *Phloeosinus* species.

### 4.1. Mating Behavior

**Figure 1:**
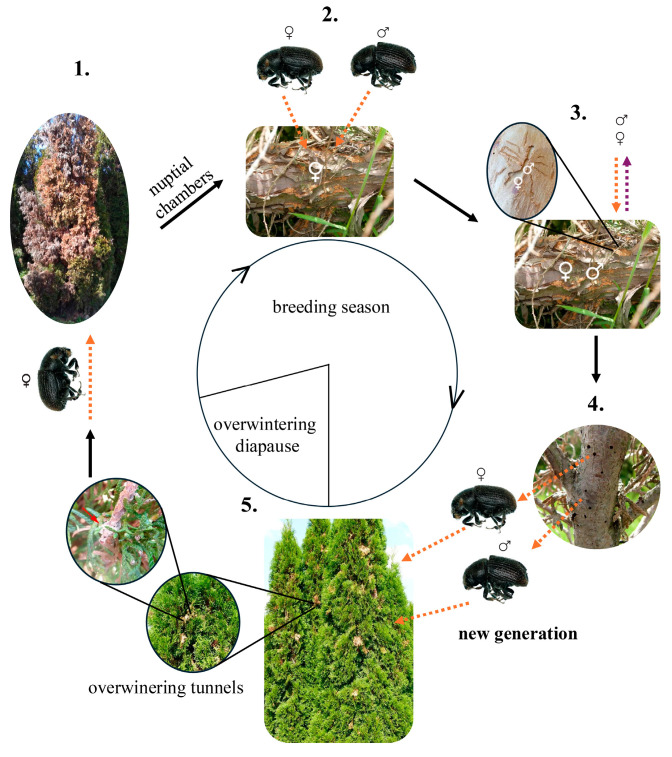
Seasonal dynamics of *Phloeosinus aubei* (Perris, 1855) galleries and breeding. The dashed arrows in phases 2 and 3 indicate the attractive (orange arrows) or repulsive effects (lilac arrows) of semiochemicals released by conspecifics. The semiochemical background is described in Table 2 [45,60,61].

In *P. aubei*, the female is the pioneer sex in host colonization. After leaving their overwintering tunnels, the females select weakened or stressed Cupressaceae trees in which they build the nuptial chambers, a preference that is supported by field observations from different regions, including the eastern Mediterranean and the temperate zone of Europe, indicating that colonization is typically restricted to physiologically compromised or fungus-infested host trees [31,45]. Then, females attract males for mating (see Section 4.3). Afterward, they construct brood galleries (Figure 1). This sequence, in which females determine the primary colonization site and males arrive secondarily, resembles the typical reproductive behavior of *Dendroctonus* species [59].

Semiochemicals play a crucial role in host selection (see Section 4.2), mate attraction and host colonization (see Section 4.3). Beyond that, mating behavior in *P. aubei* is not limited to pheromonal communication. Short-range vibrational signals are decisive for mate recognition and courtship after the males arrive on the host tree [45,60]. This multimodal communication, involving plant kairomones, beetle pheromones, and physical interactions between the sexes ensures that mating occurs only after resources for reproduction have been secured.

### 4.2. Host Plant Kairomones

Volatiles of Cupressaceae are central for the host colonization strategy of *P. aubei*. Females use plant odors to discriminate vulnerable weak trees from healthy trees [45,62]. For example, studies using gas chromatography coupled with electroantennographic detection (GC-EAD) revealed that they perceive 22 components emanating from *Thuja occidentalis*. The most abundant of these components were the oxygenated monoterpenes, α- and β-thujone, fenchone, camphor, terpinen-4-ol, bornyl acetate and α-terpinyl acetate [60]. Comparative analyses of volatiles from healthy and declining trees showed that the emission of α-pinene was markedly greater in the latter. Furthermore, reduced emissions of α-thujone and fenchone and increased emissions of β-pinene and myrcene were observed in the volatiles of weakened host trees compared to healthy ones [62].

Electroantennographic (EAD) studies on *P. aubei* using pure enantiomers as cues have shown that α- and β-thujone, (+)-β-pinene, (−)- and (+)-terpinen-4-ol and *cis*-4-thujanol elicit the strongest antennal responses in both sexes, while (−)- and (+)-camphor elicit stronger response in females than in males [63,64].

While there is only moderate EAD activity of (−)- and (+)-α-pinene, laboratory four-arm olfactometer and field bioassays have demonstrated that both sexes of *P. aubei* are strongly attracted to synthetic blends of the two enantiomers. This suggests that α-pinene is a key compound from declining trees that serves as a kairomone for host selection [60,61,62].

*Phloeosinus aubei* is not the only xylophagous pest of Cupressaceous trees. The cypress jewel beetle *Ovalisia* (*Palmar*) *festiva* (Linnaeus, 1767) (Coleoptera: Buprestidae) is also specialized to the same host species. Moreover, these two pests often compete to infest the same trees. However, GC-EAD studies suggest that the two species use different plant volatiles for host selection and perceive certain compounds differently. For example, (*E*)-β-caryophyllene elicited an antennal response only in *O. festiva*, whereas borneol elicited a response only in *P. aubei*. Of the substances detected by both, *P. aubei* shows the strongest antennal responses to (+)-β-pinene and α- and β-thujone. In *O. festiva* the strongest responses were evoked by (−)- and (+)-terpinen-4-ol and (+)-fenchone [64].

It has long been recognized that *P. aubei* often colonizes host trees suffering from cypress canker. This fungal disease is caused by *Seiridium* spp., (Ascomycota: Sordariomycetes: Amphisphaeriales: Sporocadaceae), typically by *S. cardinale* (W.W. Wagener) B. Sutton & I.A.S. Gibson [31,65,66]. Although this association is frequent, the causal link between the pest and the pathogen still needs to be clarified. Similarly, it has not yet been studied whether the volatile profile of *Seiridium* infested trees differs from that of healthy trees or trees stressed by other factors.

Several studies have demonstrated that *P. aubei* responds strongly to host-derived volatiles, which act as kairomones guiding beetles to suitable conifer hosts. Sun et al. [67] reported that α-pinene was the dominant compound in branch and foliage volatiles, while thujopsene dominated trunk volatiles, and 3-carene was detected in both tissues. Field assays revealed species-specific responses: *P. aubei* was significantly attracted to a blend of turpentine oil and ethanol, whereas the sympatric *P. hopehi* Schedl, 1953 responded primarily to high-release-rate α-pinene. Hayes et al. [68] showed that *P. scopulorum* Swaine, 1924, and *P. serratus* (LeConte, 1868) were strongly attracted to ethanol-baited juniper trees, and attraction was further enhanced by a combination of berry oil, cade oil, and ethanol. These results indicate that ethanol can serve as a proxy for host stress volatiles and elicits strong attraction in field assays, although its natural production by the host and role as a true kairomone remain uncertain [68]. Yang et al. [69] demonstrated in laboratory bioassays that both male and female *P. aubei* adults were attracted to essential oils from stressed *Platycladus orientalis*, with attractiveness ranking as bark oils > xylem oils > leaf oils. GC-MS analysis suggested that thujopsene, 3-carene, and cedr-9-ene are likely the active components responsible for the observed attraction [69]. Fiala et al. (2024) confirmed in the field that juniper branches deployed with ethanol attracted a greater number of *P. aubei* beetles compared to other lures [70]. Collectively, these results highlight the central role of host kairomones, particularly specific host volatile compounds, in mediating host selection and aggregation behavior in *P. aubei*, while also demonstrating that ethanol is a useful experimental co-attractant for monitoring and research purposes.

### 4.3. Pheromones

The chemical communication system of *P. aubei* is based on an interplay of pheromones produced by both females and males. Pioneer females release (−)-α-pinene and (−)-myrtenol to attract mates and additional colonizers [60]. In turn, males produce (−)-α-pinene, *cis*-verbenol and verbenone [61] to initiate mass colonization and regulate attack density.

GC-EAD analyses confirmed antennal sensitivity of both sexes to the above components, identified from the hindguts of females and males, respectively [60,61]. The greatest EAG responses in males were elicited by (−)-myrtenol, (+)-*cis*-verbenol and (−)-*cis*-verbenol. Meanwhile (−)-α-pinene and (−)-verbenone elicited lower responses. In females (−)-myrtenol elicited the greatest antennal response [61].

Behavioral assays revealed their biological function as aggregation pheromones: Laboratory bioassays and field experiments using traps baited with a blend of synthetic female (−)-α-pinene and (−)-myrtenol and with male (−)-*cis*-verbenol at a ratio of 5:1:1 demonstrated that they act synergistically to attract conspecifics [61]. Adding (−)-verbenone to this blend at a 1:1 ratio relative to (−)-*cis*-verbenol reduced trap captures by 70%, proving an anti-aggregation role of this compound [61]. The experiments further yielded the best capture rates when a 7:3 mixture of (−)- and (+)-α-pinene as a host volatile was combined with the aggregation pheromone components in a ratio of 20:5:1:1 [61].

Comparisons of gut extracts of mated and unmated females revealed differences in the (−)-α-pinene to (−)-myrtenol ratio: It was found to be approximately 3:2 in unmated females, and 1:6 in mated females. The fact that the emission of these compounds depends on reproductive status supports the finding that they serve as pheromone components. This was reinforced by the treatment of unmated females with Juvenile hormone III (JHIII): Following this, the ratio increased from about 3:2 to around 5:1, which supports the endocrine regulation of α-pinene and myrtenol production [60,61].

### 4.4. Identification and Enantioselective Synthesis of Pheromone Compounds

Compounds showing electroantennographic activity in males or females were identified based on their mass spectra and retention indices. These assignments were then verified by comparison with analytical data from reference compounds.

The enantiomeric ratio of α-pinene from the hind guts of females could be determined using enantioselective GC/MS. It differed significantly from the ratio found in the host plant volatiles: While the plants produce an almost racemic mixture of enantiomers, only (−)-α-pinene was found in the female beetles. In the same way it was shown that the females selectively produce the (−)-enantiomer of myrtenol. These two female-produced compounds share an identically configured carbon skeleton. Myrtenol, verbenol and verbenone were absent from the host plant volatiles.

The reference compounds used for the identity verification, the enantioselective GC/MS-analysis and the biotest could be obtained by known efficient enantioselective synthetic procedures using commercially available (−)- or (+)-α-pinene as the starting materials.

Myrtenol ((−)- or (+)-) could be synthesized by allylic oxidation of (−)- or (+)-α-pinene with selenium dioxide in ethanol [71]. Subsequently, the respective reaction products could be enantiomerically purified through column chromatographic resolution of the diastereomeric *O*-methylmandelic esters [72].

**Figure 2:**
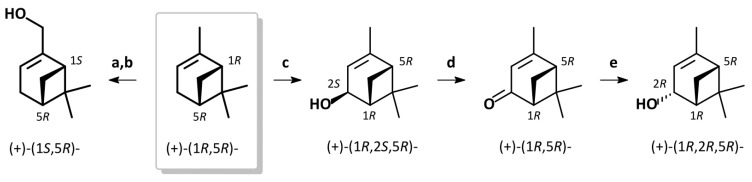

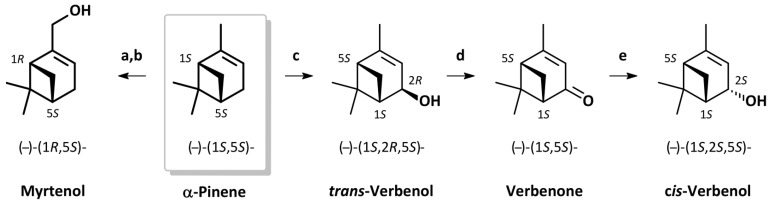
Enantioselective synthesis of α-pinene derivatives. (a) SeO_2_, EtOH, 70 °C, 2 h; NaBH_4_, EtOH, rt. (b) MPA, DCC, DMAP, DCM, 0 °C to rt 3 h; separation of diastereomers; K_2_CO_3_, MeOH, rt, 3 h. (c) Pb(OAc)_4_, benzene, 60 °C, 1 h; KOH aq, MeOH, 0 °C, 48 h. (d) Jones reagent, acetone, 0 °C, 10 min. (e) LiAlH_4_, THF, −78 °C, 2 h. MPA = (*R*)-(−)-2-methoxy-2-phenylacetic acid sense of optical rotation given for [α]_D_ (CHCl_3_).

By contrast, the allylic oxidation of (−)- or (+)-α-pinene with lead tetraacetate in benzene produced the *trans*-configurated verbenol enantiomers with retention of the carbon skeleton stereochemistry [73]. The oxidation of either (−)- or (+)-*trans*-verbenol using Jones chromic acid reagent yielded (−)-or (+)-verbenone, which could then be selectively reduced by lithium aluminum hydride (LAH) to give the corresponding *cis*-verbenol enantiomers (Figure 2) [74].

### 4.5. Biosynthetic Considerations

The colonization strategy of *P. aubei* relies on a stepwise integration of host-derived signals and beetle-produced pheromones. Initially, pioneer females locate weakened hosts using elevated emissions of α-pinene and related terpenes [44,62]. Once inside the host, females produce pheromone components, attracting males and additional females to the site [60,61]. Males, upon arrival, contribute their own pheromone signals, which not only reinforce aggregation but also introduce inhibitory compounds such as verbenone that regulate colonization density, preventing over colonization (Figure 1 and Table 2) [61].

**Figure 3:**
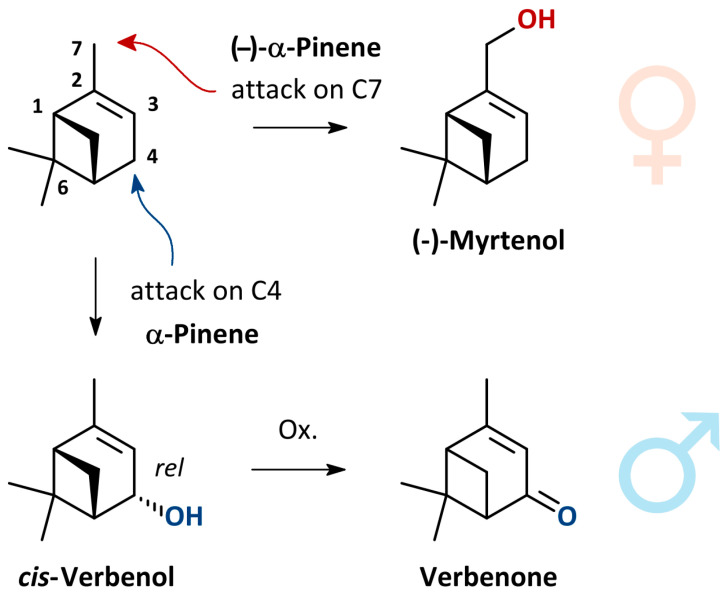
Putative biogenesis of oxygenated α-pinene derivatives as pheromone components of female and male *Phloeosinus aubei*. Absolute configuration of *cis*-verbenol and verbenone unknown. Color code refers to sexes an sex-specific functional groups in molecular formulae.

All intraspecific signaling compounds currently identified in *P. aubei* are α-pinene derivatives. Studies on the enantiomeric ratios of compounds found in females and of α-pinene in *Thuja* indicate that the oxygenated monoterpenoids are produced by the beetles from α-pinene as a common precursor. The JHIII treatment experiments support this hypothesis. Future studies determining the absolute configuration of verbenol and verbenone will seek to provide further evidence (Figure 3).

Although other bark beetle species (e.g., *Ips* and *Dendroctonus* spp. [17,75,76,77,78] are known to sequester α-pinene from their host plants and convert it into oxygenated pheromone components, the origin of α-pinene present in *P. aubei* remains unknown.

Also, the potential role of microbial endosymbionts in pheromone production is yet to be clarified.

Bioconversion processes further modulate the chemical landscape. For instance, host-emitted α-pinene can be oxidized to *cis*-verbenol and verbenone, either through beetle metabolism or microbial activity [61,75,79]. Such transformations shift the semiochemical context from attraction to inhibition, thereby preventing overexploitation of the host. This dynamic feedback mechanism, integrating kairomones, pheromones, and bioconverted derivatives, ensures efficient resource utilization and synchronized reproduction.

## 5. Conclusions

In the context of chemical ecology, the cypress bark beetle, *P. aubei*, is the most extensively studied *Phloeosinus* species. Its host choice and preference, the mating behaviour and its chemical communication have been investigated over the last decade. The preferred host plants are *T. occidentalis*, among which the beetles choose healthy trees for overwintering and declining trees for reproduction.

All stages of the colonization of *T. occidentalis* by *P. aubei* are controlled, or at least influenced, by semiochemicals: host-derived kairomones and beetle-derived pheromones.

Initially, pioneer females locate and select a suitable host tree. They respond to a characteristic bouquet of monoterpenoids emitted by *Thuja* and differentiate between weakened and healthy trees based on their altered scent profile, particularly the elevated emissions of α-pinene [45,62].

Once females have constructed the nuptial chambers, they release (−)-α-pinene and (−)-myrtenol as an aggregation pheromone to attract males and additional females. Upon arrival, males produce stridulation signals involved in short-range communication prior to mating. Meanwhile, males contribute their own pheromones: They first produce *cis*-verbenol to reinforce aggregation and finally release verbenone as an inhibitory signal to regulate colony density and prevent over-colonization (Figure 1 and Table 2) [60,61].

The structural similarity of these signaling compounds indicates a potential biosynthetic relationship. However, while structural and indirect evidence suggests a shared α-pinene-derived origin of these compounds, the biosynthetic pathways underlying pheromone production in *P. aubei*, including the potential involvement of associated microorganisms, have not yet been experimentally investigated.

From a practical perspective, the complexity of the chemical communication of *P. aubei* poses a challenge to developing semiochemical-baited traps for monitoring or pest control: Females, as pioneers, initially orient toward host-emitted kairomones. During the mass aggregation phase, attraction is enhanced by pheromones from both sexes. On the other hand, males are attracted to a mixture of host volatiles and female pheromones. However, a blend of (−)-α-pinene, (−)-myrtenol and (−)-*cis*-verbenol is more attractive to females than to males. Despite the promising results of initial efforts, trap catches still remain low near known populations, and therefore further research is needed along several lines. A critical task is to optimize the ratio of components in the multicomponent synthetic blends. This experimental approach is rather complex because the emission rate of each component will be affected by the formulation technique and the type of dispenser. Last but not least, a suitable trap design must be developed [61].

Solving these problems becomes more urgent as *P. aubei* continues to spread. It also underscores the importance of chemoecological research in the context of accelerating environmental changes.

## Figures and Tables

**Table 1:** *Phleosinus* species in Europe.

Species	Occurrence in Europe	Status in Europe	Notes/References
*Phloeosinus aubei* (Perris, 1855)	Southern, Western and Central Europe	Invasive/expanding	Native to the Mediterranean region; has expanded northwards and is considered an invasive pest of Cupressaceae (e.g., *Juniperus*, *Cupressus*, *Thuja*) in Central Europe [34,35].
*P. thujae* (Perris, 1855)	Widespread in Europe	Native	Widely distributed and considered native to Europe; associated mainly with *Thuja* and *Juniperus* spp. [24,34].
*P. rudis* Blandford, 1894	Local records in Western and Central Europe	Non-native, locally established	Native in temperate and tropical zone of Asia; introduced in Europe; sporadic records in Western Europe, often linked to ornamental Cupressaceae [34,36].
*P. armatus* Reitter, 1894	Southern Europe (e.g., Greece, Italy, Cyprus), introduced in Russia	Native/invasive	Native to the eastern Mediterranean region; invasive status described from Russia [24,37,38].
*P. cedri* Brisout de Barneville, 1883	Southwestern Europe (e.g., Spain)	Native (Mediterranean)	Associated mainly with *Cedrus* spp.; limited distribution in Europe [39,40].
*P. gillerforsi* Bright, 1987	Azores	Endemic/localized	Known only from the Azores and Canary Islands; not invasive [39].
*P. laricionis* Faccoli & Sidoti, 2013	Italy	Native (local)	Rare species with restricted distribution [27].
*P. pfefferi* Knížek, 1994	Cyprus	Native (local)	Endemic or near-endemic to Cyprus [41].
*P. sequoiae* (Hopkins, 1903)	Germany	Non-native/ expanding	Native in North America, the first European record was described from north-western Germany [42].
*P. henschi* Reitter, 1901	Bulgaria, Italy, Greece, Croatia, Ukraine	Native	Associated with pathogenic fungi (*Geosmithia* spp.) [39,43].

**Table 2:** Semiochemicals of *Phloeosinus aubei* and their effects correspond to the breeding phase. The numbers refer to the breeding phase in Figure 1.

Semiochemicals for *Phloeosinus aubei*	No. of Phase in Figure 1	Releaser	Role	Description
(−)- and (+)-α-pinene	1.	host	kairomone	It is a key component of host colonisation. It is produced in increased amounts by a decaying host [60,62].
(−)-α-pinene	2. and 3.	female and male	aggregation pheromone	It is produced by both male and female *P. aubei*. It is a precursor of the gender-specific pheromone components [60,61].
(−)-myrtenol	2.	female	aggregation pheromone	It is produced by the pioneer gender (female) and attracts both sexes of conspecifics [60].
*cis*-verbenol	3.	male	aggregation pheromone	It is produced when males are joined to females [61].
verbenone	3.	male	anti-aggregation pheromone	It has an anti-aggregation, repellent effect for both sexes [61].

## Data Availability

No new data were created or analyzed in this study.
